# Grapheme-Color Synesthesia and Its Connection to Memory

**DOI:** 10.7759/cureus.67524

**Published:** 2024-08-22

**Authors:** Stefani Anash, Andrew Boileau

**Affiliations:** 1 School of Medicine, Saba University School of Medicine, The Bottom, BES; 2 Neurology, Saba University School of Medicine, The Bottom, BES

**Keywords:** sensation, synesthesia, memory, declarative memory, grapheme-color synesthesia

## Abstract

Synesthesia is the involuntary association of different senses, where individuals experience one sensory modality in response to the stimulation of another. For example, a synesthete may perceive colors when reading certain numbers or associate specific tastes with particular words. Synesthesia manifests differently for individuals grouping the condition in subcategories such as grapheme-color, sound-to-color, lexical-gustatory, mirror-touch, and much more. This review covers grapheme-color synesthesia, described as the involuntary perception of specific colors or color associations when seeing or thinking about certain letters, numbers, or symbols. This review explores the performance of declarative memory tasks in individuals with grapheme-color synesthesia. A comprehensive search of controlled trials published between 2014 to 2024 was conducted through PubMed and Google Scholar databases. In Google Scholar, the search terms grapheme-color synesthesia, grapheme-color synaesthesia, and memory were used. In PubMed, additional MeSH (Medical Subject Headings) terms were used which included grapheme-color synesthesia and memory. Studies that measured declarative memory and grapheme-color synesthesia were included yielding a total of seven controlled trials. Grapheme-color synesthetes demonstrated advanced performance in declarative memory tasks; however, this may not have any clinical significance. Grapheme-color synesthetes demonstrated a better performance in their ability to recall colors, but not as much recalling words. Synesthetes were shown to outperform non-synesthetes in visual memory tasks. Synesthetes showed better recall of paired patterns, shape-color associations, and visual grids compared to control groups, but the influence of synesthesia on word memory remains unclear. Future research should consider adding control for confounding factors, collaborating with other institutions, and increasing sample size.

## Introduction and background

"Synesthesia", or “synaesthesia”, has its origin in the Greek roots, syn, meaning union, and aesthesis, meaning sensation referring to the union of the senses. According to the most accurate study of the prevalence of synesthesia, it affects 4.4% of the world population [1]. It is the involuntary association of different senses, where individuals experience one sensory modality in response to the stimulation of another. Synesthesia manifests differently for individuals, grouping the condition into about 60 different subcategories [2]. Examples include grapheme-color, mirror-touch, lexical-gustatory, and several more. Grapheme-color synesthesia involves the associations of colors of certain letters or numbers. Synesthetes in this category involuntarily perceive specific colors or color associations when they see or think about certain letters, numbers, or symbols. For example, some grapheme-color synesthete will say the number six is orange or the letter D is brown. The assignment of color to a number or letter is subjective and can vary between individuals. Mirror-touch synesthesia is when one watches another person being physically touched and feels as though they are being touched. Lexical-gustatory synesthesia involves tasting certain words when hearing or reading those words. Synesthesia uniquely blends sensory perceptions creating a diverse number of subtypes. 

Synesthesia can be traced back to the mid-1700s when famous poet and philosopher Johann Gottfried Herder documented his confusing experience with the condition. In 1848, a French physician Charles-Auguste-Édouard Cornaz published a medical dissertation describing synesthesia as the opposite of color blindness. The term “hyperchromatopsie” laid a foundation for what would eventually be termed synesthesia [3]. Famous synesthete in the 1920s, Solomon Shereshevsky, recalled lists of digits, numbers, street names, and more in a matter of minutes. His synesthesia quickly rose to attention when he was able to memorize anything he was told by using visual mnemonics. He was followed for 30 years by neurophysiologist Alexander Luria who first suggested in his case study that synesthesia may be linked to better memory [4]. 

There is no widely accepted explanation of the physiology behind synesthesia and theories are continuously expanding. One theory suggests that grapheme-color synesthesia can be associated with an increase in grey matter in the left angular gyrus [5]. Another theory suggests that grapheme-color synesthesia may develop during childhood while learning colors and letters. When doing so, children start to associate the two and take those associations with them into adulthood which eventually develops into grapheme-color synesthesia [6]. Hancock et al. support this theory by stating the association of colors and letters can be attributed to the colors of letter magnets commonly used on refrigerators in North America [7]. Children learn colors and letters at the same time causing them to associate the two. Another theory suggests that individuals with synesthesia have hyperconnectivity between different sensory regions of the brain [8]. The cross-activation theory similarly states that there is cross-activation in different processing areas of the brain including area V4 (associated with color perception) and the inferior temporal cortex [9].

Although it is not considered to be a clinical disorder, there have been questions as to whether synesthesia might be either beneficial or detrimental to learning and memory. Van Leeuwen et al. suggest that synesthesia is linked to autism spectrum disorder because of hypersensitivity to senses which interferes with daily living [10]. On the other hand, Bremer et al. argue that most individuals consider their synesthesia as a gift that does not interfere with their daily living, but rather enriches it [11]. The type of synesthesia should also be considered when answering this question. Although it has been reported that synesthesia can enrich senses and creativity, in a case study with a person with reverse word synesthesia, the patient reported her synesthesia to be debilitating rather than enriching [12]. Her form of synesthesia caused her to impulsively flip words backward whether she was reading or talking. 

This review specifically considers grapheme-color synesthesia and declarative memory. Declarative memory covers the recall of facts through long-term memory. While the mechanism behind synesthesia has been widely studied since the 1900s, the link between synesthesia and memory did not start being studied until the early 2000s when case studies looked at synesthetes individually and tested their memory. A case study by Smilek et al. tested an individual in her memory abilities to recall colored digits in which she performed near-perfectly [13]. Studies by Yaro et al. started gathering larger subject groups and had participants perform memory tasks further suggesting that synesthetes might have better memory than non-synesthetes [14]. Eventually, studies started to connect the idea that individuals with grapheme-color synesthesia may perform better than the general population in declarative memory tasks. This paper aims to delve deeper into the intricacies of synesthesia by exploring its connection to declarative memory.

## Review

Methods

A comprehensive search was conducted through two databases: PubMed and Google Scholar. In Google Scholar, the search terms "grapheme-color synesthesia" AND/OR "grapheme-color synaesthesia" AND "memory" were used. In PubMed, additional MeSH terms were used which included (grapheme-color synesthesia[MeSH Terms]) AND (memory[MeSH Terms]).

**Figure 1:**
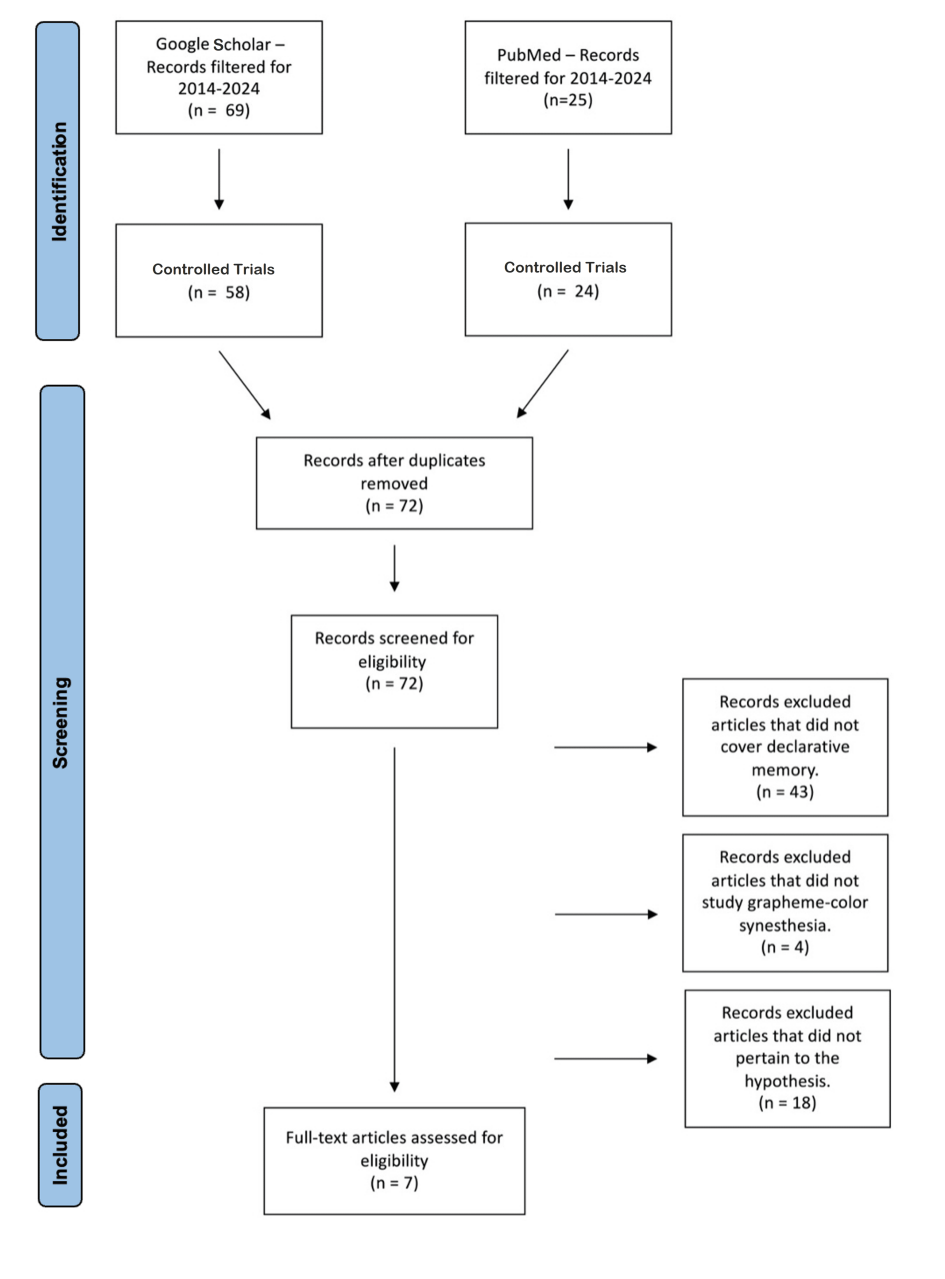
PRISMA-style flow diagram PRISMA: Preferred Reporting Items for Systematic Reviews and Meta-Analyses

Both databases were searched using filters “past 10 years” and controlled trials only, yielding 58 records from Google Scholar and 24 from PubMed. Records from both databases were combined and 10 duplicates were removed yielding in a total of 72 results. Of those 72 results, each article was individually screened for eligibility. A total of 43 records were excluded for not measuring declarative memory. Four records were excluded for not studying grapheme-color synesthesia. Additionally, 18 articles were excluded that did not pertain to the hypothesis in other ways. In total, there were seven articles that were eligible for analysis (Figure 1).

Results

For the selection process in this review, seven papers fit the inclusion and exclusion criteria. All primary papers found were controlled trials using various memory tests as their research design. Some of these studies demonstrated an advantage to declarative memory recall in those with grapheme-color synesthesia depending on the type of memory being tested.

Recruited from the United Kingdom Synesthesia Association website via the University of Sussex, Pfeifer et al. compared grapheme-color synesthetes to two different control groups separated into young adults and older adults [15]. The young adult control group (n=14) was aged 19-29 years (M=22.64) with eight females. The older adult control group (n=14) was aged 62-83 years (M=68.79) with nine females. The synesthesia group (n=14) had an age range of 19-31 years (M=22.50) with nine females. Participants were given three pairs of black-and-white photos with similar patterns and five pairs of dissimilar patterns. The three groups were instructed to memorize the eight total pairs. After learning the paired pattern associations, participants were presented with one picture of the pairs and were required to match them to their matching photo.

The difference in results was analyzed through analyses of covariance (ANCOVA) through partial eta squared (η_p_^2^). The accuracy of paired pattern recall of each picture was observed and the measurement of accuracy was defined as the hit rate. The average dissimilar hit rate for synesthetes was the highest at 81.48 ± 1.54 (mean ± SD) and the lowest in the older adult group (67.22 ± 2.53). The control young adult group fell in between the two (79.45 ± 1.90). The number of times required by each participant to learn a full set of eight pair associates is defined as the number of runs through each round. Older adults had the greatest number of runs (7.93 ± 1.23) while synesthetes had the lowest number of runs (3.21 ± 0.30). Young adults fell in the middle with the number of runs (3.21 ± 0.30). The increased number of runs had a significant effect on the contribution to the hit rate for dissimilar pairs (p<0.001, ηp2 = 0.307). In recalling the three similarly paired pictures, the number of runs did not significantly predict the hit rate (p = 0.124, ηp2 = 0.061). Hit rates were high in all three groups showing no difference (Young adults: 96.87 ±1.40, older adults: 91.23 ± 3.83, and synesthetes: 98.93 ± 0.73) [15].

The design of Bankieris and Aslin's study consisted of shape-color pairings [16]. Participants included a total of 14 self-reported synesthetes (26.7±11.6, mean±SD, four males) and a total of 15 non-synesthetes (20.1 ± 3.0, seven males). The study design comprised a three-by-three grid with nine various white snowflake shapes along with nine randomly assigned colors with each respective snowflake shape. In the pretest phase, participants were given the nine snowflake shapes and asked for any previously associated colors. This method was used to solidify the control group in the study. Subjects who reported associating a specific color with a snowflake were further analyzed to be put in the synesthetes group rather than the control group. The learning phase and testing phases were divided into seven blocks. During the learning phase, participants were presented with a three-by-three grid of white snowflakes. Touching each snowflake on the touchscreen computer allowed participants to learn their respective associated colors assigned by the study. After 36 touches, the learning phase automatically ended and was quickly followed by the testing phase. In this phase, each white snowflake shape was presented individually on a screen and participants had to choose its assigned color via a color picker and luminescence slider. Participants were asked to return two weeks later to go through the same testing phase to assess long-term memory.

Bankieris and Aslin analyzed the results via a “mixture model analysis” to determine if participants were able to accurately pick color and shade based on memory. The level of significance of the hypothesis being tested was defined as 𝛼. Correctly choosing the color of the target with variability was defined as 𝛼_TS_ for synesthetes and 𝛼_TC_ for the control group. Choosing the color of a nontarget item with variability was defined as 𝛼_NS_ for synesthetes and 𝛼_NC_ for the control group. Randomly guessed colors were defined as 𝛼_RS_ for synesthetes and 𝛼_RC_ for the control group. Through this mixture model analysis, it was found that the synesthesia group was able to reproduce the hue of colors more accurately than the control group (𝛼_TS_ = 0.77, 𝛼_TC_ = 0.54, p < .001) and fewer nontarget responses than controls (𝛼_NS_ = 0.06, 𝛼_NC_ = 0.36, p < .001). Participants demonstrated similar results two weeks later with the synesthesia group reproducing the hue of colors more accurately than the control group (𝛼TS = 0.88, 𝛼TC = 0.77, p < .01) and a trend toward fewer nontarget responses than controls (𝛼_NS_ = 0.02, 𝛼_NC_ = 0.07, p = 0.06). The overall analysis of this study demonstrated that synesthetes learn and recall shape-color at a higher and faster advantage in both the short term and long term [16].

In an autobiographical memory recall study, Chin and Ward recruited 44 grapheme-color synesthetes (mean age=40.4 years, SD=14.5; 25 females) and 40 non-synesthetes (mean age=43.6 years, SD=11.6; 39 females) [17]. Both groups participated in an online survey where they were presented with six neutral cue words: chocolate, train, doctor, snow, vacation, and party. Participants were then asked to describe detailed memories from two distinct time periods: early childhood (ages 0-12 years old) and a recent timeframe ranging from three months to three years ago, for each cue word. The description had to be specific including all senses they could remember. Following their description, they were asked to answer 14 questions involving each sense. Senses were broken down into four subsections. The recollection section involved five questions covering details, sound, sight, reliving, and emotion. The belief section involved questions on spatial awareness, coherence, time, confidence, and agreement that they saw the event with their own eyes and if the event really occurred. The rehearsal section asked questions regarding whether they had thought of this memory before the study or if it came to them out of the blue. The last section asked the subject how this memory impacts them.

Synesthetes were able to report a more recollective experience as compared to their control counterparts (p < 0.001, η_p_^2^ = 0.232). Synesthetes demonstrated that they were able to preserve their remote childhood memories more than non-synesthetes (p < 0.001, η_p_^2^= 0.544). Non-synesthetes were shown to have forgotten more from their childhood. Because the time period between present time and childhood varies for different age groups, age played a factor in the data. Effects of enhanced childhood memories in synesthetes were not affected by age (p > 0.100). Whether the participant was younger or older, the sharpness of childhood memories remained unchanged. A difference was found in rehearsal where non-synesthetes reported better recent memory recall (p = 0.004) while synesthetes showed better childhood memory recall (p = 0.026) [17].

Simner and Bain tested memory recall advantage in five randomly sampled 10-11-year-old children with grapheme-color synesthesia [18]. Participants were recruited from screenings performed in previous years by the research group. Synesthetes were compared to a control group of 40 participants with the same mean age. The control group was broken up into 20 children categorized as having high memory and 20 categorized as having average memory. The letter-span task measured the ability to hold verbal information for a short time period known as the phonological loop function. Participants were verbally told a series of letters and asked to immediately recall given letters verbally at a pace of one letter per second. The letter matrix task tested the same five synesthetes and 10 high memory and 10 low memory controls. In this test, subjects were instructed to view a four-by-four grid filled with letters for one minute, after which the screen went black, and they were required to fill in a paper copy with as many letters as they could remember with no time limit.

Accuracy and speed of recall were measured in the letter-span task and performance was analyzed via ANOVA. There were six trials and letters ranged up to nine. Participants had to correctly recall four of the six trials to proceed. After three incorrect trials, the testing stopped, and the maximum letter-span set was measured. The higher the letter-span number represented memory advantage. Children with grapheme-color synesthesia showed a non-significant trend of memory advantage over the average memory control (p=0.06) and showed no difference in comparison to the high-memory controls (p = 0.7). In the letter-matrix task, data showed that there was no significant difference between the three groups (p = 0.7). Entering data into a Bayes analysis gave a Bayes factor of 0.18 (a Bayes factor less than 0.33 shows strong support for the null hypothesis) [18].

Lunke and Meier recruited 52 grapheme-color synesthetes and matched them to 52 controls according to age, gender, and level of education [19]. Synesthetes were sourced from the Synesthesia-Check website created by the program. Volunteers were given 24 words they had to memorize in addition to 19 filler words such as days and months. In the study phase, they were given each word on a screen one at a time and were required to pick one of 13 colors that they believed were associated with that word. One hour later, volunteers were presented with old and new words and had to categorize them respectively. The accuracy of organization tested the participants’ short-term memory.

To assess general short-term memory performance, results were analyzed via a two-by-four-by-three ANCOVA and showed no main effect for synesthesia (p = 0.441, η_p_^2^ = 0.01). Between synesthesia and type of stimuli, an interaction occurred (p = 0.002, η_p_^2^= 0.12). Age, used as a covariate, showed a main effect but did not interact (p < 0.001, η_p_^2 ^= 0.26). Post-hoc tests showed that grapheme-color synesthetes had an advantage for color stimuli (p = 0.012, d = 0.63) and for music (p = 0.049, d = 0.46) and a disadvantage for words (p = 0.036, d = -0.50) [19].

Grapheme-color synesthetes showed memory advantage in correct recall of given words defined as the z-transformed Pr scores for each type of synesthesia. Memory advantage was present in grapheme-color synesthetes when given color stimuli (0.51±0.16, mean±SD) as opposed to their control counterparts (0.45±0.13). Performance was consistent across different colors. However, the presence or absence of color stimuli did affect performance. When presented with words alone without a color stimulus, synesthetes showed little difference in memory advantage (0.47±0.26) as compared to the control group (0.58±0.16; p > 0.05) [19].

To assess recollection of words, Lunke and Meier conducted repeated measures of ANCOVA for recollection. The study looked for interactions due to the influence of different variables. An effect was found for synesthesia (MSE = 0.05, p = 0.021, η_p_^2^ = 0.03). However, no main effect was found for the type of synesthesia (MSE = 0.05, p = 0.725, η_p_^2^ < 0.01). There was no interaction found between the two (MSE = 0.05, p = 0.207, η_p_^2^ = 0.02). There was a significant interaction between the type of synesthesia and the type of stimuli (MSE = 0.03, p = 0.012, η_p_^2^≤ 0.05). Age, as a covariate, also showed a significant main effect (MSE = 0.05, p < 0.001, η_p_^2^= 0.11) and interacted with type of stimuli (MSE = 0.03, p = 0.004, η_p_^2^ = 0.03). In the posthoc tests, grapheme-color synesthetes presented higher recollection for colors (p = 0.044, d = 0.47) and lower recollection for words (p = 0.04, d =-0.49) [19].

In their first experiment, Mealor et al. recruited 17 older synesthetes with an average age of 64 years (SD = 6.91) and 29 control non-synesthetes with an average age of 67 years old (SD = 6.09) [20]. In the young group, there were 22 synesthetes with an average age of 23 years (SD = 3.39) and 20 controls with an average age of 23 (SD = 2.81). Grapheme-color synesthetes were confirmed via a standardized test on the synesthete.org website. In a random order, participants were given memory stimuli including three-digit numbers, images of snowflakes, and short musical passages. Each module had a training phase in which they were instructed to memorize the stimuli and a testing phase. The testing phase presented the stimuli and compared them to a new or old set of stimuli. Participants were instructed to click response keys if they perceived the given stimuli as old or new.

The overall performance or accuracy was measured by d-prime in a two-by-two-by-three ANOVA. Individually, there showed to be a significant effect of age (η_p_^2^ = 0.096, p=0.002) and synesthesia (η_p_^2^ = 0.150, p<0.001) on memory. Performance of both young and old grapheme-color synesthetes showed higher d-prime scores than controls. Young synesthetes showed a d-prime score of 0.88 with a standard error of the mean as 0.06. The young control group showed a d-prime score of 0.61 with a standard error of the mean of 0.05. The older synesthetes group showed a d-prime score of 0.69 with a standard error of the mean of 0.07. The older control group showed a d-prime score of 0.50 with a standard error of mean of 0.05 [20]. 

There was no significant interaction between age and synesthesia (η_p_^2^ = 0.007, p=0.424). Because there was no evidence of a correlation between the two, this study showed that synesthesia does not allow for a protective effect on age-related memory decline. There was a main effect of modality (p < 0.001, η_p_^2^ = 0.087) and an interaction between age and (p < 0.001, η_p_^2^ = 0.121) with the musical stimuli showing the largest age-related loss. No other interactions were significant (p > 0.1). Bayes factor (BF) was used to further investigate the lack of statistical significance between age and synesthesia. The Bayes factor was found to be 0.25, providing evidence against the theory that synesthesia has protective effects on memory decline. The authors concluded that the memory enhancement associated with synesthesia does not influence memory decline in aging [20].

In their second experiment, Mealor et al. recruited 18 older synesthetes aged 65±4.52 years and 21 older controls aged 67±5.63 years [20]. They also recruited 18 young synesthetes (age 23±3.01 years) and 84 young controls (22±2.50 years). Grapheme-color synesthetes were confirmed in the same manner as the first experiment. In this experiment, subjects were instructed to memorize a five-by-two grid in the learning phase. The grid contained shapes and colors along the grid and it stayed up throughout the whole learning phase. While the grid remained within sight, 10 stimuli appeared on the computer screen for three seconds each. Similarly to the first experiment, during the testing phase, participants were given some old and new shapes and colors along the grid and were instructed to identify them. Participants identify old as “correct” and new as “incorrect”. This tested shape, color, and location.

Overall performance was measured using d-prime and a two-by-nine-by-two ANOVA showed a significant main effect of synesthetes outperforming non-synesthetes (p = 0.033, η_p​​​_​​​​^2^ = 0.033). There was also a significant main effect of younger people outperforming older people (p = 0.014, η_p_^2^ = 0.043. Synesthesia and age did not show significant interaction (p = 0.573, η_p_^2^ = 0.002). Bayes factor calculation shows evidence in agreement with the null hypothesis stating there is no significance between synesthesia and its protective effects on memory decline (BF = 0.41). The study measured color, shape, and location performance individually by using d-prime in a three-by-two-by-two mixed ANOVA. Significance was seen with the main effects of age (p = 0.002, η_p_^2^ = 0.067), and synesthesia (p = 0.005, η_p_^2^= 0.055). The interaction between age and synesthesia was not significant (p = 0.351, η_p_^2^= 0.006). Significance was seen with the effect of stimulus (p < 0.001, η_p_^2^= 0.346). Stimulus and age showed a significant interaction (p = 0.022, η_p_^2^= 0.030). There were also significant interactions between stimulus and synesthesia (p = 0.035, η_p_^2^= 0.024). There was no significance in interactions between stimulus, age, and synesthesia together (p = 0.198, η_p_^2^= 0.012) [20].

Lunke and Meier studied grapheme-color synesthesia and its role in recognition memory through retaining words and colors individually [21]. The study design consisted of 19 grapheme-color synesthetes (17 female and two male) and 79 controls recruited via the Synesthesia-Check website from the University of Bern. The mean age for synesthetes is 46.79 years (SD=18.19) and 48.21 years (SD=18.03) for the control group. The study was broken down into two phases: the study phase and the recognition phase. In the word study phase, participants were instructed to read a list of words on the screen, one at a time. They were told to select a color that was best associated with each word. In the color study phase, participants were given different color patterns and had to study them for three seconds each. After that, they rated how much they liked the patterns on a seven-point scale. During the word recognition phase, participants were given a mix of new words and words used in the study phase, and they had to recognize whether the words were old or new. In the color recognition phase, participants were presented with old and new color patterns and had to identify which one was old and new. The test was conducted through two sessions which were one year apart.

The results of their study showed grapheme-color synesthetes had a longer memory advantage because they had a smaller forgetting score as compared to the control group. The forgetting score was used in both the word and color recognition phases. Using a two-by-three ANOVA showed a significant effect of synesthesia (p = 0.048, MSE = 0.06, η_p_^2^ = 0.11) and a significant effect of type of stimuli (p < 0.001, MSE = 0.03, η_p_^2^ = 0.36) but no interaction between the two (p = 0.492, MSE = 0.03, η_p_^2^ = 0.02). Between the first and second sessions, grapheme-color synesthetes had a forgetting score significantly smaller for color recall (p = 0.051, Cohen’s d = −0.55), and words (p = 0.045, Cohen’s d = −0.57), whereas the decay for music was not significant (p = 0.477, d = −0.24). Overall, the study showed a smaller forgetting score for colors rather than words supporting grapheme-color synesthetes’ long-lasting memory with colors specifically [21].

Table 1 shows a summary of the findings of the included studies.

**Table 1:** Summary of study findings

TpFirst Author	Objective	Study Design		Level of Evidence	Study Population	Therapy or Exposure	Results Summary
Bankieris and Aslin (2016) [16]	To assess whether synesthetes demonstrate superior short-term memory tasks in recalling snowflake-color pairings.		Control Trial	2	14 synesthetes and 15 non-synesthetes from the Rochester area	Snowflake color pairings	Synesthetes learned snowflake-color pairings more quickly than controls. Synesthetes demonstrated better color recall than their peers.
Chin and Ward (2017) [17]	To study childhood memory recall in synesthetes and non-synesthetes.		Control Trial	2	44 grapheme-color synesthetes and 40 non-synesthetes in the University of Sussex	Autobiographical memory tests.	Synesthetes reported sharper childhood memories as compared to non-synesthetes reported more vivid memories from adulthood
Lunke and Meier (2018) [19]	To assess memory recall in grapheme-color synethetes when given color stimuli.		Control Trial	2	52 grapheme-color synesthetes and matched them to 52 controls according to age, gender, and level of education	Color and word recognition in random order.	The results showed a memory benefit with color stimuli in those with grapheme-color synesthesia.
Lunke and Meier (2020) [21]	To evaluate long-term recall in grapheme-color synesthetes compared to their control counterpart.		Control Trial	2	19 grapheme- color synesthetes recruited via the synesthesia-check on the website of the University of Bern and 76 healthy control-participants matched for age, gender and education participated.	Recognition memory tests.	The advantage for memorizing color shown by grapheme-color synesthetes was persistent after one year compared to their matched controls.
Mealor et al. (2019) [20]	To evaluate protective effects of memory in grapheme-color synesthetes compared to non-synesthetes.		Control Trial	2	17 older synesthetes, 29 older control, 22 young synesthetes, 20 control	Recognition memory tests.	Grapheme-color synesthesia does not have a protective effect against memory decline in aging
Pfeifer et al. (2014) [15]	To compare memory advantage in grapheme-color synesthetes compared to non-synesthetes in young and old age groups.		Control Trial	2	14 young synesthetes, 14 young, and 14 older adults.	Visual associative learning computer program.	The results show a subtle associative memory advantage in synesthetes for non-synesthesia inducing stimuli, which can be detected against older adults.
Simner and Bain (2018) [18]	To evaluate enhanced abilities in performing tasks in synesthetes compared to their control sounterpart.		Control Trial	2	Group of randomly sampled child synesthetes age 10 and 11 years.	Cognitive memory tests.	Synesthetes demonstrated above-average performance in a processing-speed task and a near-significant advantage in a letter-span task.

Discussion

Every study focused on the short-term memory of participants and one study additionally included a follow-up test to assess long-term memory one year later. All the studies involved experiments focused on testing memory. Examples included tasks such as memorizing and recalling words, pictures, grids, or a combination of different stimuli.

The studies that used pictures for the memory studies were by Pfeifer et al. [15] and Bankieres and Aslin [16]. Pfeifer et al. gave participants eight pairs of different black-and-white photos [15]. Three of those pairs posed pictures that were similar but not similar enough to be obvious. They were tested on their visual memory and observed based on age and the presence or absence of grapheme-color synesthesia. Overall, the study found a memory advantage in grapheme-color synesthetes over their control counterparts. Although they presented with an advantage, it was not enough to be seen with the young controls. Advanced memory performance was more apparent when synesthetes were compared to older control participants suggesting the performance was not enough to consider synesthesia as an advantage to memory. Synesthetes may use their synesthesia as a mnemonic tool, but it would not be enough to set them apart from non-synesthetes. Bankieries and Aslin conducted a similar study but with black-and-white snowflake shape pairs on a three-by-three grid [16]. The study concluded that grapheme-color synesthetes learn and recall visual images better and faster both short and long term. Although synesthetes performed better at memory tasks, age was not considered a confounding factor in their study. This suggests that these memory tasks may appear impressive, but it is unclear how they may translate to everyday situations. 

Similar to how Bankieries and Aslin [16] used a three-by-three grid to conduct their studies, this type of methodology was popular among other experiments such as those by Simner and Bain [18] and Mealor et al. [20]. The second experiment done by Mealor et al. involved memorizing a five-by-two grid filled with shapes and colors [20]. When instructed to recall the grids, participants had to recognize whether they were presented with those specific shapes and colors before categorizing them as “new” or “old”. The study showed that there was no significance between synesthesia and its protective effects on memory decline. This can suggest that there is no relation between memory and synesthesia, or that synesthesia’s impact on memory is more of a slight enhancement than a significant advantage. Simner and Bain had a study design containing random letters on a four-by-four grid they called their letter matrix test [18]. They required participants to memorize the grids for one minute and rewrite them on a blank sheet of paper. Their study found that grapheme-color synesthetes performed better and faster in memory tasks than their control counterparts. Because of the small sample size, results were trending but statistically insignificant. Additionally, the study design could be questioned as being too simple: memorizing a four-by-four grid is relatively straightforward. Developing more advanced memory tests and increasing sample size would be required to get more accurate results. 

In addition to grid tests, Simner et al. conducted letter span tasks [18]. After being verbally presented with a sequence of letters, participants were required to orally recall as many of those letters as they could. Although grapheme-color synesthetes performed better than average memory controls, there was no difference between synesthetes and high memory controls. Grapheme-color synesthetes showed an advantage to visual learning. It could be less advantageous for them to complete tasks involved in auditory learning. Participants in Lunke and Meier's 2018 study were also asked to memorize words but were allowed to associate words with a color of their choice giving them a grapheme-color stimulus [19]. When they were presented with various words, they were asked to identify them as “old” or “new” providing grapheme-color synesthetes with the opportunity to stimulate their synesthesia through choosing color associations with given words. They demonstrated a significant advantage in their memory performance. Further research is needed to explore this phenomenon, specifically focusing on how stimuli can trigger and enhance the memory advantage in synesthetes.

Autobiographical memory was tested by Chin and Ward who gave participants different cue words to determine if it triggered memories differently in grapheme-color synesthetes compared to non-synesthetes [17]. Memories from adulthood to childhood were analyzed in subjects. Synesthetes reported sharper childhood memories as compared to non-synesthetes who reported more vivid memories from adulthood. There was no definitive explanation as to why childhood memories seemed to be clearer. However, one explanation is that the vibrant colors of toys and cartoons during childhood may trigger synesthesia. On the other hand, it can be argued that synesthesia may be learned through associations based on exposure in childhood. It raises the question of whether synesthesia is a learned phenomenon. Synesthetes may have learned to associate senses but is unclear why some people experience it and some do not. 

Some studies used mixed stimuli tests to see how it affects memory in grapheme-color synesthesia. Mealor et al. used a mixed stimulus of three-digit numbers, images of snowflakes, and musical passages in their first experiment [20]. Subjects were instructed to memorize the three stimuli that were presented in a consistent order. After studying various quantities of stimuli, they were presented with new and previously seen stimuli and were required to identify them as old or new. The study found that synesthesia does not have a protective effect against memory decline in aging again suggesting there may not be a connection between synesthesia and memory.

Common limitations

Several limitations can arise in studying grapheme-color synesthesia irrespective of the design of the study. Self-reported synesthesia is a common limitation across all studies. A significant proportion of the population may not be aware of their synesthesia. Participants in the studies who are aware of their synesthesia may introduce bias to the study. They may consciously create their own synesthetic mnemonics during studies potentially leading to skewed results. Memory performance can be affected by other factors outside of synesthesia such as education level, general intelligence levels, social factors, etc. The outcome of results can also be attributed to the Hawthorne effect in which participants may have increased or decreased performance in memorizing since they all knew they were being studied. Blinded studies should be considered in the future. Additionally, synesthesia is not a linear experience. Although all subjects here reportedly had grapheme-color synesthesia, they may not experience it in the same way. Limitations arise in this review structure as well. There were only seven studies that met the criteria. Further reviews should expand the criteria. The variability in types of studies also poses a limitation in the consistency of results. Finally, a majority of the primary articles included in this review were conducted by the same or overlapping research groups. Seven authors from these primary articles are based at the University of Sussex, which could possibly introduce bias into their experiments. It is crucial to diversify research on grapheme-color synesthesia to reduce potential bias. Collaborations should be done with other institutions to ensure wider viewpoints to extend research.

## Conclusions

This review examined how grapheme-color synesthesia can affect declarative memory performance. Synesthetes showed to outperform non-synesthetes in visual memory tasks. Synesthetes showed better recall of paired patterns, shape-color associations, and visual grids compared to control groups. The influence of synesthesia, either grapheme-color or other subtypes, on memorizing words remains unclear. Future research should address limitations identified in this review such as controlling for confounding factors, sample size and selection, and standardized testing.

## References

[1] Simner J, Mulvenna C, Sagiv N (2006). Synaesthesia: the prevalence of atypical cross-modal experiences. Perception.

[2] Banissy MJ, Jonas C, Cohen Kadosh R (2014). Synesthesia: an introduction. Front Psychol.

[3] Jewanski J, Simner J, Day SA, Rothen N, Ward J (2020). The evolution of the concept of synesthesia in the nineteenth century as revealed through the history of its name. J Hist Neurosci.

[4] Mecacci L (2013). Solomon V. Shereshevsky: the great Russian mnemonist. Cortex.

[5] Arend I, Yuen K, Sagi N, Henik A (2018). Neuroanatomical basis of number synaesthesias: a voxel-based morphometry study. Cortex.

[6] Watson MR, Akins KA, Spiker C, Crawford L, Enns JT (2014). Synesthesia and learning: a critical review and novel theory. Front Hum Neurosci.

[7] Hancock P (2013). Synesthesia, alphabet books, and fridge magnets. Oxford Handbook of Synesthesia.

[8] Maurer D, Ghloum JK, Gibson LC (2020). Reduced perceptual narrowing in synesthesia. Proc Natl Acad Sci U S A.

[9] Hubbard EM, Brang D, Ramachandran VS (2011). The cross-activation theory at 10. J Neuropsychol.

[10] van Leeuwen TM, Wilsson L, Norrman HN, Dingemanse M, Bölte S, Neufeld J (2021). Perceptual processing links autism and synesthesia: a co-twin control study. Cortex.

[11] Bremer Bremer, J J (2015). Mental disorder or creative gift? The cognitive scientific approach to synesthesia. Forum Philos.

[12] Osuagwu FC, Plath D (2017). A case of reverse word synesthesia in a young woman. Prim Care Companion CNS Disord.

[13] Smilek D, Dixon MJ, Cudahy C, Merikle PM (2002). Synesthetic color experiences influence memory. Psychol Sci.

[14] Yaro C, Ward J (2007). Searching for Shereshevskii: what is superior about the memory of synaesthetes?. Q J Exp Psychol (Hove).

[15] Pfeifer G, Rothen N, Ward J, Chan D, Sigala N (2014). Associative memory advantage in grapheme-color synesthetes compared to older, but not young adults. Front Psychol.

[16] Bankieris KR, Aslin RN (2016). Explicit associative learning and memory in synesthetes and nonsynesthetes. Iperception.

[17] Chin T, Ward J (2018). Synaesthesia is linked to more vivid and detailed content of autobiographical memories and less fading of childhood memories. Memory.

[18] Simner J, Bain AE (2018). Do children with grapheme-colour synaesthesia show cognitive benefits?. Br J Psychol.

[19] Lunke K, Meier B (2018). New insights into mechanisms of enhanced synaesthetic memory: benefits are synaesthesia-type-specific. PLoS One.

[20] Mealor AD, Simner J, Ward J (2020). Does synaesthesia protect against age-related memory loss?. J Neuropsychol.

[21] Lunke K, Meier B (2020). A persistent memory advantage is specific to grapheme-colour synaesthesia. Sci Rep.

